# The significance of *p53* codon 72 polymorphism for the development of cervical adenocarcinomas

**DOI:** 10.1054/bjoc.2001.2085

**Published:** 2001-10

**Authors:** S Andersson, E Rylander, A Strand, J Sällström, E Wilander

**Affiliations:** Department of Obstetrics and Gynaecology, Huddinge University Hospital, (Affiliated with the Karolinska Institute), Huddinge, 14186, Sweden; Department of Obstetrics and Gynaecology, Danderyd Hospital, Danderyd, 18288, Sweden; Department of Medical Sciences, Dermatology and Venereology, University Hospital, Uppsala, 75185, Sweden; Department of Genetics and Pathology, Section of Clinical Cytology, University Hospital, Uppsala, 75185, Sweden

**Keywords:** cervix, adenocarcinomas, HPV-infection, p53, polymorphism

## Abstract

Infection with the human papillomavirus is an important co-factor in the development of cervical carcinomas. Accordingly, HPV DNA is recognised in most of these tumours. Polymorphism of the *p53* gene, codon 72, is also considered a risk factor in the development of cervical carcinoma. However, this finding is contradicted by several observers. In the present investigation, 111 cases of adenocarcinoma of the cervix collected through the Swedish Cancer Registry and 188 controls (females with normal cytology at organised gynaecological screening) were analysed with regard to p53, codon 72, polymorphism using a PCR- and SSCP-based technique. In the controls, 9% showed pro/pro, 44% pro/arg and 47% arg/arg, whereas in the invasive adenocarcinomas, the corresponding figures were 0%, 29% and 71%, respectively. The difference was statistically significant (*P* = 0.001). HPV DNA was identified in 86 tumours (HPV 18 in 48, HPV 16 in 31 and HPV of unknown type in 7 cases) and 25 tumours were HPV negative. The p53, codon 72, genotypes observed in HPV-positive and HPV-negative cervical adenocarcinomas were not statistically different (*P* = 0.690). The results indicate that women homozygotic for arg/arg in codon 72 of the *p53* gene are at an increased risk for the development of cervical adenocarcinomas. However, this genetic disposition seems to be unrelated to the HPV infection. © 2001 Cancer Research Campaign  http://www.bjcancer.com


*British Journal of Cancer* (2001) **85**(8),1153–1156
© 2001 Cancer Research Campaign
doi: 10.1054/ bjoc.2001.2085, available online at http://www.idealibrary.com on IDEAL

### The significance of *p53* codon 72 polymorphism for the development of cervical adenocarcinomas

**S Andersson[1], E Rylander[2], A Strand[3], J Sällström[4] and E Wilander[4]**

[1]Department of Obstetrics and Gynaecology, Huddinge University Hospital, (Affiliated with the Karolinska Institute) 14186 Huddinge, Sweden; [2]Department of Obstetrics and Gynaecology, Danderyd Hospital, 18288 Danderyd, Sweden; [3]Department of Medical Sciences, Dermatology and Venereology, University Hospital, 75185 Uppsala, Sweden; [4]Department of Genetics and Pathology, Section of Clinical Cytology, University Hospital, 75185 Uppsala, Sweden

**Summary** Infection with the human papillomavirus is an important co-factor in the development of cervical carcinomas. Accordingly, HPV DNA is recognised in most of these tumours. Polymorphism of the *p53* gene, codon 72, is also considered a risk factor in the development of cervical carcinoma. However, this finding is contradicted by several observers. In the present investigation, 111 cases of adenocarcinoma of the cervix collected through the Swedish Cancer Registry and 188 controls (females with normal cytology at organised gynaecological screening) were analysed with regard to p53, codon 72, polymorphism using a PCR- and SSCP-based technique. In the controls, 9% showed pro/pro, 44% pro/arg and 47% arg/arg, whereas in the invasive adenocarcinomas, the corresponding figures were 0%, 29% and 71%, respectively. The difference was statistically significant ($P$ = 0.001). HPV DNA was identified in 86 tumours (HPV 18 in 48, HPV 16 in 31 and HPV of unknown type in 7 cases) and 25 tumours were HPV negative. The p53, codon 72, genotypes observed in HPV-positive and HPV-negative cervical adenocarcinomas were not statistically different ($P$ = 0.690). The results indicate that women homozygotic for arg/arg in codon 72 of the *p53* gene are at an increased risk for the development of cervical adenocarcinomas. However, this genetic disposition seems to be unrelated to the HPV infection. © 2001 Cancer Research Campaign http://www.bjcancer.com

**Keywords**: cervix; adenocarcinomas; HPV-infection; p53; polymorphism

Cervical carcinoma is the second most common type of cancer in females worldwide, with over 500 000 new cases diagnosed each year. However, the incidence is declining in developed countries owing to established cytological screening programmes.

In most cancer registries, close to 75% of the tumours are reported to be of the squamous type. Adeno- and adenosquamous carcinomas represent about 10–15% and invasive tumours with unspecified histology constitute the remaining 10–15% (Paloma et al, 1998).

It is now widely accepted that the human papillomavirus (HPV) is the major causal factor in most, if not all cervical cancers. This fact makes cervical cancer probably the most common virally caused cancer (Zur Hausen, 1991).

However, the prevalence of HPV in squamous carcinoma is almost 100% (Walboomers et al, 1999), but in cervical adenocarcinoma, it is only about 70%, according to recent reports (Iwasowa et al, 1996; Paloma Vizcaino et al, 1998).

HPV 16 is the most commonly detected genotype in pre-invasive and invasive squamous carcinoma (50–60%), followed by HPV 18 which is present in about 15% of the tumours (Zehbe and Wilander, 1997a). However, in cervical adenocarinoma, HPV 18 is the predominating type of virus followed by HPV 16 (Tenti et al, 1996).

Molecular biological studies have revealed that a polymorphism at codon 72 of the human tumour-suppressor gene, *p53*, results in translation to either arginine or proline. The risk of the development of cervical cancer associated with the human papillomavirus is considered higher for women homozygous for arginine than for those who are heterozygous. (Storey et al, 1998). However, this observation is both confirmed and contradicted by several groups (Hildesheim-A et al, 1998; Josefsson et al, 1998; Rosenthal et al, 1998; Zehbe et al, 1999; Agorastos et al, 2000; Makni et al, 2000). The aim of the present investigation was to examine polymorphism in *p53*, codon 72, in women with invasive cervical adenocarcinomas and to compare the results with those of previous studies performed on patients with pre-invasive and invasive cervical squamous carcinomas. Furthermore, we intended to examine whether the p53 polymorphism was correlated with an HPV infection in the tumours.

*Received 12 March 2001*
*Revised 19 July 2001*
*Accepted 1 August 2001*

*Correspondence to:* S Andersson

### MATERIAL AND METHODS

### Tumour material

A collection of cervical adenocarcinomas was used for the study. Tumour blocks were obtained from different Departments of Pathology, with the aid of the Cancer Registry of the National Board of Health and Welfare in Sweden. The material has previously been described in detail (Andersson et al, 2001).

The original collection consisted of formalin-fixed (tumour blocks) from 180 women which were sectioned and stained with haematoxylin-eosin for light microscopic analysis. From the light microscopic examination and the women's medical records, 23 tumours were considered to be of non-cervical origin, 14 tumours were classified as squamous carcinomas and 9 as adenosquamous carcinomas. 131 relevant cases, selected after microscopic analysis and evaluation of the medical records of the women, were further used. However, due to a lack of sufficient material for DNA extraction or a negative β-globin test (see below) an additional

20 cases had to be excluded. The remaining 111 cases were used for HPV tests and the examination of p53, codon 72, polymorphism.

### DNA extraction

DNA was isolated in one 10-μm thick section from each selected tissue block (Lungu et al, 1992). The sections were treated for 3 hours with 500 μg $ml^{-1}$ proteinase K in 150 μl digestion buffer (50 mM KCl, 10 mM Tris-HCl, pH 8.3) at 65˚C. The enzyme was then inactivated by boiling for 10 min.

### HPV-testing

The HPV tests were performed as described earlier (Zehbe et al, 1996; Zehbe and Wilander, 1997). Briefly, the analyses were performed on extracted DNA (Lungu et al, 1992) obtained from sections of the paraffin blocks of which the preceding section had been used for morphological diagnosis. In the first sequence, the availability of tissue DNA was examined by performing a β-globin test (Saiki et al, 1988). Secondly, the extracted DNA was analysed with the SHARP Signal System (Digene Diagnostics, Beltsville, MD) (Manos et al, 1989; Zehbe and Wilander, 1997) using MY09/MY11 primers from the L1 gene of HPV.

Specimens showing HPV of a high-risk type were subjected to HPV typing (HPV of a low-risk type was not observed in our material). HPV DNA from the L1 region was amplified with GP5+/GP6+ primers and the amplicon products were HPV-typed by single-strand conformational polymorphism (SSCP). The method has previously been described in detail (De Roda Husman et al, 1995; Zehbe et al, 1996).

To optimise the HPV PCR analysis, all HPV-negative adenocarcinomas were subjected to a second HPV test using specific primers from the E6 genes of HPV 16 and 18 for the PCR reaction (Alemi et al, 1999) (Table 1). Thereafter, PCR products, indicating infection with HPV 16 or 18, were identified on agarose gels.

### *p53* polymorphism test

The *p53* exon 4 PCR reaction was performed with two 21 bp primers (Table 1), and at an annealing temperature of 62˚C resulting in a 155-bp reaction product.

For p53 genotyping, single-strand conformation polymorphism (SSCP) was used, employing the Gene Gel Excel 12.5 kit (Pharmacia Biotech, Uppsala, Sweden). The PCR products were run on a GenePhor Electrophoresis Unit at 400 V, 25 mA, 12˚C for 100 min. After separation, the 12.5% polyacrylamide gel was silver-stained in a Hoefer Automated Gel Stainer together with the Plus One DNA Silver Staining kit (Figure 1).

DNA preparations obtained from cytobrush specimens in the 188 normal females (25–60 years old), participating in the organised gynaecological screening in the county of Uppsala, were used as controls in the p53, codon 72 genotype study.

### Statistical analysis

The $\chi^2$ test for trend was used for the statistical comparison of the proportions (Breslow and Day, 1980).

### RESULTS

Of the 131 adenocarcinomas collected for the study, a successful β-globin test and a *p53*, codon 72 analysis were obtained in 111 tumours. They were of the papillary or glandular type and lacked a squamous component.

### HPV analysis

By using the MY09/MY11 primers from the *HPV L1* gene for the PCR reaction and the SHARP Signal System, 77/111 (69%) of the tumours were determined HPV positive. With the application of the specific HPV 16 E6 and HPV 18 E6 primers on the remaining HPV negative tumours, an additional 9 cases 86/111 (77%) showed HPV DNA, of which HPV 16 was identified in 4, and HPV 18 in 5 tumours, respectively. In the HPV positive adenocarcinomas, 48/86 (43%) were infected with HPV 18, 31/86 (31%) with HPV 16 and the remaining 7/86 (7%) with other or unknown types of HPV. 25 (23%) cases were HPV negative (Table 2).

### p53 analysis

The 3 *p53*, codon 72, genotypes arg/arg, arg/pro and pro/pro all gave divergent band patterns with the PCR-SSCP analysis as illustrated in Figure 1.

**Table 1** Primers used for PCR

| Primer pair | Sequence 5′–3′ | Position of gene | Length (bp) | Product size (bp) |
|---|---|---|---|---|
| β-globin/PCO4 | CAACTTCATCCACGTTCACC | 54–73 | 20 | 268 |
| β-globin/GH20 | GAAGAGCCAAGGACAGGTAC | 195–176 | 20 | |
| HPV/MY09 | CGTCCMARRGGAWACTGATC[a] | L1 (HPV6) | 20 | 398 |
| HPV/MY11[b] | GCMCAGGGWCATAAYAATGG[a] | | 20 | |
| HPV/GP5+ | TTTGTTACTGTGGTAGATACTAC | L1 (HPV16) | 23 | 141 |
| HPV/GP6+ | GAAAAATAAACTGTAAATCATATTC | L1 (HPV16) | 25 | |
| HPV 16 | CTAAAATTAGTGAGTATAGACATTA | E6 | 25 | 176 |
| HPV 16 | CCTTATATTATGGAATCTTTGC | E6 | 22 | |
| HPV 18 | ATGTTGCCTTAGGTCCATGCA | E6 | 21 | 570 |
| HPV 18 | ACCGAAAACGGTCGGGACC | E6 | 19 | |
| p53, codon 72 | GACCCAGGTCCAGATGAAGCT | Exon 4 | 21 | 155 |
| p53, codon 72 | ACCGTAGCTGCCCTGGTAGGT | Exon 4 | 21 | |

HPV, Human papillomavirus.
[a]Degenerate code, M = A + C; R = A + G; W = A + T; X = G + C.
[b]Biotinylated at its 5′ end.

 *© 2001 Cancer Research Campaign*

**Table 2** Prevalence of various genital types of HPV identified in the cervical adenocarcinomas

| Cervical adenocarinomas | Number of cases | (%) |
|---|---|---|
| HPV-18 pos | 48 | 43 |
| HPV-16 pos | 31 | 28 |
| HPV pos, other types | 7 | 6 |
| HPV neg | 25 | 23 |
| Total | 111 | |

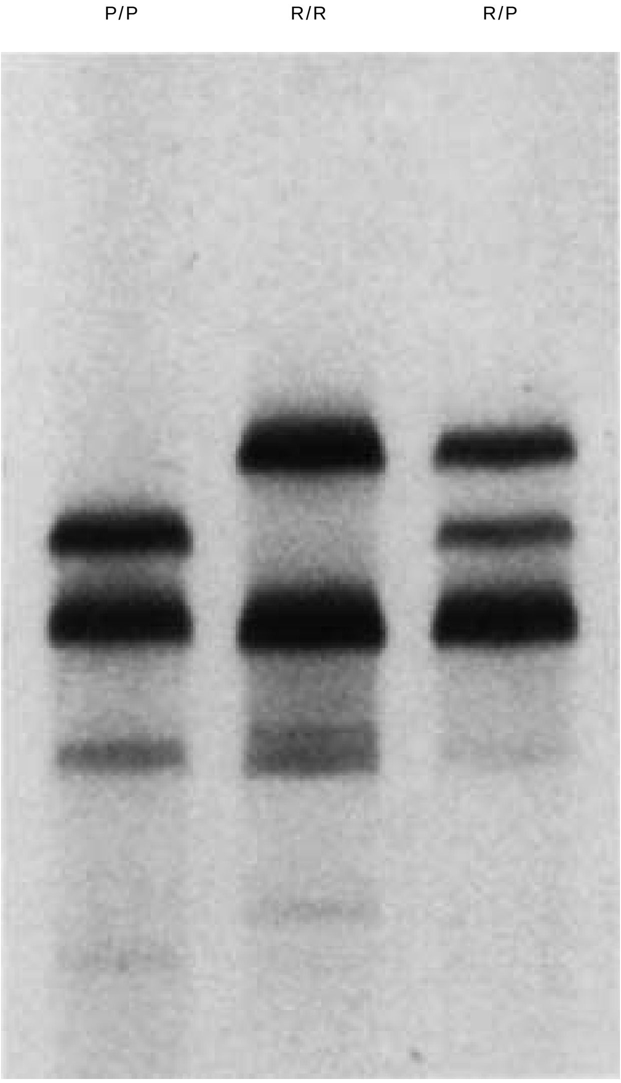

**Figure 1** PCR-SSCP genotyping of the p53 codon 72 polymorphism. The lanes show women homozygotic for pro/pro (lane 1), heterozygotic for arg/pro (lane 3) and homozygotic for arg/arg (lane 2), respectively

In the 188 normal female controls, the distribution of genotypes was arg/arg 47%, arg/pro 44% and pro/pro 9%. The corresponding figures for the cervical adenocarcinomas were arg/arg 71%, arg/pro 29% and pro/pro 0%. The difference between normal controls and women with cervical adenocarcinomas was statistically highly significant ($P < 0.001$). However, the small difference obtained between HPV-positive and HPV-negative tumours was not statistically significant ($P = 0.690$) (Table 3).

**Table 3** p53, codon 72, genotypic distribution in normal controls and HPV positive and negative cervical adenocarcinomas

| | Pro/Pro (%) | Pro/Arg (%) | Arg/Arg (%) | Total |
|---|---|---|---|---|
| Normal controls | 16 (9) | 83 (44) | 89 (47) | 188 |
| Adenocarcinomas | | | | |
| HPV-18 pos | 0 (0) | 15 (31) | 33 (69) | 48 |
| HPV-16 pos | 0 (0) | 7 (23) | 24 (77) | 31 |
| HPV-X pos | 0 (0) | 2 (29) | 5 (71) | 7 |
| HPV neg | 0 | 8 (32) | 17 (68) | 25 |
| Total | 0 | 32 (29) | 79 (71) | 111 |

### DISCUSSION

The prevalence of HPV observed and the distribution of the different types of HPV that we identified in the cervical adenocarcinomas are in general agreement with previous observations of these tumours (Iwasawa et al, 1996; Paloma Vizcaino et al, 1998). These findings emphasise the existence of some pathogenetic difference in comparison to the cervical squamous carcinomas, which are almost always HPV positive and where HPV 16 predominates over HPV 18. Recently, it was shown that women with HPV positive adenocarcinomas are younger than those with HPV negative adenocarcinomas (Tenti et al, 1996; Andersson et al, 2001). Moreover, this observation indicates that carcinogenic factors other than HPV are involved in the development of at least some cervical adenocarcinomas.

Since the original report by Storey et al (Storey et al, 1998) that women with the *p53* codon 72 arg/arg genotype are at an increased risk of developing cervical carcinomas, a series of publications has occurred dealing with this enigma. The results of several investigations are concordant with those of Storey et al (Storey et al, 1998; Zehbe et al, 1999), whereas several others are unable to support the finding (Minaguchi et al, 1998; Klaes et al, 1999; Brooks et al, 2000). All these studies are performed on squamous carcinomas or tumours without defined histological typing.

In the present report of pure cervical adenocarcinomas, we found a significant increase ($P = 0.001$), of the arg/arg in comparison with normal controls. Thus, in Swedish women, adenocarcinomas and squamous carcinomas of the cervix seem to manifest identical behaviour, since in a previous investigation in Sweden, women with the arg/arg genotype were correlated to an increased risk of developing squamous carcinoma (Zehbe et al, 1999). The distribution of *p53* codon 72 genotypes, which we found in the normal female population, is equivalent to that observed in a previous study of Swedish women (Zehbe et al, 1999). However, the prevalence of the different *p53*, codon 72, genotypes is unequally distributed in several countries and therefore the importance of the arg/arg genotype in the development of cervical cancer is of variable significance (Tenti et al, 1998). Furthermore, since the *p53* polymorphism was not distributed differently among HPV-positive and -negative adenocarcinomas, it is suggested that the increased risk of cervical adenocarcinoma in arg/arg women is not directly related to the HPV infection. Our results further support the opinion that polymorphism in the human genoma, at a critical site, can affect the rate of incidence of cancer (Rosenthal et al, 1998; Storey et al, 1998). In addition, single-point mutations in the DNA of oncogenic types of HPV can have a corresponding biological potential. For instance, an amino acid shift in codon 83 of the E6 protein of HPV 16 Leu<Val seems to increase both the persistence

*© 2001 Cancer Research Campaign*

of infection and the malignant properties of the virus in Swedish women (Zehbe et al, 1998; Andersson et al, 2000).

Apparently, the interplay between the human genotype and oncogenic variants of HPV are of importance for the risk of the development of cervical cancer. However, the evaluation of this interplay is complicated by the normal variation of the human genoma in different geographic regions and by an uneven distribution of the types and variants of HPV in the world (Koutsky, 1997). The introduction of gynaecological screening, about 30 years ago, has been successful in reducing the rate of incidence of squamous carcinoma of the cervix in the developed countries (Möhlck et al, 1994; Bergström et al, 1999). However, during the same time period, the rate of incidence of adenocarcinoma of the cervix has increased, especially in younger women (Miller et al, 1993). Evidently, cytological screening has not been able to reduce the incidence of adenocarcinoma. The introduction of HPV testing, an increased knowledge of the importance of polymorphism in the human genoma for the development of tumours and continued research on the oncogenic properties of the different types and variants of HPV are considered as instrumental in reducing the incidence of cervical adenocarcinoma.

### ACKNOWLEDGEMENTS

We wish to thank Sonja Andersson and Margit Gustafsson for technical assistance. The statistical analysis of Marit Degerman and the photographic work of Frank Bittkowski are greatly appreciated. This study was supported by the Swedish Cancer Foundation, the Karolinska Institutet Foundation, Stockholm and the Medical Faculty of the Uppsala University Sweden.

### REFERENCES

Agorastos T, Lambropoulos AF, Constantinidis TC, Kotsis A and Bontis JN (2000) p53 codon 72 polymorphism and risk of intra-epithelial and invasive cervical neoplasia in Greek women [In Process Citation], *Eur J Cancer Prev* **9**: 113–118

Alemi M, Andersson S, Sällström J and Wilander E (1999) Rapid test for identification of human papillomavirus 16 E6 L83V variant. *Diagn Mol Pathol* **8**: 97–100

Andersson S, Alemi M, Rylander E, Strand A, Larsson B, Sällström J and Wilander E (2000) Uneven distribution of HPV 16 E6 prototype and variant (L83V) onkoprotein in cervical neoplastic lesions. *British J of Cancer* **83**: 307–310

Andersson S, Rylander E, Larsson B, Strand A, Silfversvärd and Wilander E (2001) The role of human papillomavirus in cervical adenocarcinoma carcinogenesis, *European Journal of Cancer* **37**: 246–250

Bergström R, Sparén P and Adami HO (1999) Trends in cancer of the cervix uteri in Sweden following cytological screening. *Br J Cancer* **81**: 159–166

Breslow NE and Day NE (1980) Statistical methods in cancer research. *International Agency for Research on Cancer* **1**: 129–144

Brooks L, Tidy J, Gusterson B, Hiller L, O'Nions, Gasco M, Marin MC, Farrell P, Kaelin W and Crook T (2000) Preferential retention of codon 72 arginine p53 in squamous cell carcinomas of the vulva occurs in cancers positive and negative for human papillomavirus. *Cancer Research* **60**: 6875–6877

De Roda Husman A-M, Walboomers JMM, van den Brule AJC, Meijer CJLM and Snijders PJF (1995) The use of the general primers GP5 and GP6 elongated at their 3′ ends with adjacent highly conserved sequences improves human papillomavirus detection by PCR. *J Gen Virol* **76**: 1057–1062

Hildesheim-A, S-M, Brinton-LA, Fraumeni JR-JF, Herrero R, Bratti-MC et al (1998) p53 polymorphism and risk of cervical cancer, *Nature* **396**: 531

Iwasawa A, Nieminen P, Lehtinen M and Paavonen J (1996) Human papillomavirus DNA in uterine cervix squamous cell carcinoma and adenocarcinoma detected by polymeras chain reaction. *Cancer* **77**: 2275–2279

Josefsson AM, Magnusson PK, Ylitalo N, Quarforth-Tubbin P, Ponten J, Adami HO and Gyllensten UB (1998) p53 polymorphism and risk of cervical cancer [letter; comment], *Nature* **396**: 531; discussion 532

Klaes R, Ridder R, Schaefer U, Benner A and von Knebel Doeberitz M (1999) No evidence of p53 allele-specific predisposition in human papillomavirus-associated cervical cancer, *J Mol Med* **77**: 299–302

Koutsky L (1997) Epidemiology of genital human papillomavirus infection. *Am J Med* **102**: 3–27

Lungu O, Wright TC and Silverstein S (1992) Typing of human papillomavirus by polymerase chain reaction amplification with L1 consensus primer RFLP analysis. *Mol Cell Probes* **6**: 145–152

Mählck CG, Jonsson H and Lenner P (1994) Pap smear screening and changes in cervical cancer mortality in Sweden. *Int J Gynaecol Obstet* **44**: 267–272

Makni HFE, Kaiano J et al (2000) p53 polymorphism in codon 72 and risk of HPV-induced cancer: effect of inter-laboratory variation, *International Journal of Cancer*, **87**: 528–533

Manos MM, Ting Y, Wright DK, Lewis AJ, Broker TR and Wolinsky SM (1989) Use of polymerase chain reaction amplification for the detection of genital human papillomaviruses. *Cancer Cells* **7**: 209–214

Miller BE, Flax SD, Arheart K and Photopulos G (1993) Human Papillomavirus types 16 and 18 infection in infiltrating adenocarcinoma of the uterine cervix. *Cancer* **72**: 1281–1285

Minaguchi T, Kanamori Y, Matsushima M, Yoshikawa H, Taketani Y and Nakamura Y (1998) No evidence of correlation between polymorphism at codon 72 of p53 and risk of cervical cancer in Japanese patients with human papillomavirus 16/18 infection, *Cancer Res* **58**: 4585–4625

Paloma Vizcaino A, Moreno V, Bosch Xavier F, Munoz Nubia, Barros-Dios XM and Parkin DM (1998) International trends in the incidence of cervical cancer: 1. Adenocarcinoma and adenosquamous cell carcinomas. *Int J Cancer* **75**: 536–545

Rosenthal AN, Ryan A, Al-Jehani RM, Storey A, Harwood CA and Jacobs IJ (1998) p53 codon 72 polymorphism and risk of cervical cancer in UK. *Lancet* **352**: 871–872

Saiki RK, Chang CA, Levenson CH, Warren TC, Boehm CD, Kazaian HH et al (1988) Diagnosis of sickle cell anemia and β-thalassemia with enzymatically amplified DNA and non-radioactive allele-specific oligonucleotide probes. *N Engl Med* **319**: 537–541

Storey A, Thomas M, Kalita A, Harwood C, Gardiol D, Mantovani F, Breuer J, Leigh IM, Matlashewski G and Banks L (1998) Role of a p53 polymorphism in the development of human papillomavirus-associated cancer [see comments], *Nature* **393**: 229–234

Tenti P, Romagnoli S, Silini E, Zappatore R, Spinillo A, Giunta P et al (1996) Human Papillomavirus types 16 and 18 infection in infiltrating adenocarcinoma of the cervix. *Anatom Pathol* **106**: 52–56

Tenti P, Pavanello S, Padovan L, Spinillo A, Vesentini N, Zappatore R et al (1998) Analysis and clinical implications of p53 gene mutations and Human Papillomavirus type 16 and 18 infection in primary adenocarcinoma of the uterine cervix. *Am J Pathology* **152**: 1057–1063

Walboomers JM, Jacobs MV, Manos MM, Bosch FX, Kummer JA, Shah KV, Snijders PJ, Peto J, Meijer CJ and Munoz N (1999) Human papillomavirus is a necessary cause of invasive cervical cancer worldwide. *J Pathol* **189**(1): 12–19

Zehbe I, Evander M, Edlund K, Rylander E, Wadell G and Wilander E (1996) Non-isotopic detection and typing of human papillomavirus (HPV) by use of polymerase chain reaction and single strand conformation polymorphism (PCR-SSCP). *Am J Surg Pathol Part B: Diagn MoPathol* **5**: 206–213

Zehbe I and Wilander E (1997a) Human papillomavirus infection and invasive cervical neoplasia: a study of prevalence and morphology. *J Pathol* **181**: 270–275

Zehbe I and Wilander E (1997b) Nonisotopic ELISA-based detection of human papillomavirus-amplified DNA. *Mod Pathol* **10**: 188–191

Zehbe I, Wilander E, Delius H and Tommasino M (1998) Human Papillomavirus 16 E6 variants are nore prevalent in invasive cervical carcinoma than the prototype, *Cancer Research* **58**: 829–833

Zehbe I, Voglino G, Wilander E, Genta F and Tommasino M (1999) Codon 72 polymorphism of p53 and its association with cervical cancer [letter], *Lancet* **354**: 218–219

zur Hausen H (1991) Human papillomaviruses in the pathogenesis of anogenital cancer. *Virology* **184**: 9–13

 *© 2001 Cancer Research Campaign*
